# Evolutionary patterns and processes in the radiation of phyllostomid bats

**DOI:** 10.1186/1471-2148-11-137

**Published:** 2011-05-23

**Authors:** Leandro R Monteiro, Marcelo R Nogueira

**Affiliations:** 1 Department of Biological Sciences and Hull York Medical School, The University of Hull, Hull, HU6 7RX, UK; 2 Laboratorio de Ciencias Ambientais, Universidade Estadual do Norte Fluminense, Av. Alberto Lamego 2000, Campos dos Goytacazes, RJ, Brazil; 3 Departamento de Biologia Animal, Universidade Federal Rural do Rio de Janeiro, Seropedica, RJ, Brazil

## Abstract

**Background:**

The phyllostomid bats present the most extensive ecological and phenotypic radiation known among mammal families. This group is an important model system for studies of cranial ecomorphology and functional optimisation because of the constraints imposed by the requirements of flight. A number of studies supporting phyllostomid adaptation have focused on qualitative descriptions or correlating functional variables and diet, but explicit tests of possible evolutionary mechanisms and scenarios for phenotypic diversification have not been performed. We used a combination of morphometric and comparative methods to test hypotheses regarding the evolutionary processes behind the diversification of phenotype (mandible shape and size) and diet during the phyllostomid radiation.

**Results:**

The different phyllostomid lineages radiate in mandible shape space, with each feeding specialisation evolving towards different axes. Size and shape evolve quite independently, as the main directions of shape variation are associated with mandible elongation (nectarivores) or the relative size of tooth rows and mandibular processes (sanguivores and frugivores), which are not associated with size changes in the mandible. The early period of phyllostomid diversification is marked by a burst of shape, size, and diet disparity (before 20 Mya), larger than expected by neutral evolution models, settling later to a period of relative phenotypic and ecological stasis. The best fitting evolutionary model for both mandible shape and size divergence was an Ornstein-Uhlenbeck process with five adaptive peaks (insectivory, carnivory, sanguivory, nectarivory and frugivory).

**Conclusions:**

The radiation of phyllostomid bats presented adaptive and non-adaptive components nested together through the time frame of the family's evolution. The first 10 My of the radiation were marked by strong phenotypic and ecological divergence among ancestors of modern lineages, whereas the remaining 20 My were marked by stasis around a number of probable adaptive peaks. A considerable amount of cladogenesis and speciation in this period is likely to be the result of non-adaptive allopatric divergence or adaptations to peaks within major dietary categories.

## Background

The Phyllostomidae (leaf-nosed bats) is the dominant family of bats in Central and South America. This family has undergone an adaptive radiation unparalleled among other mammals in terms of ecological and morphological diversity [1]. Starting from an insectivore ancestor in the late Eocene [2-4], the 53 extant genera in this family have diversified into specialized forms for insectivory, carnivory, frugivory, granivory, nectarivory, and sanguivory (although many species have mixed diets) [5-7]. This ecological diversity and specialisation originates from an intricate partitioning of resources [8,9], and is probably responsible for the high local species richness (ranging between 31-49 syntopic species [10,11]) observed for leaf-nosed bats. The evolution of specialised diets created functional demands, apparently determining changes in cranial and mandibular shape [1,12]. The magnitude of skull form (shape + size) variation among phyllostomid lineages is large and has been assessed by correlational studies both qualitatively and quantitatively, using measurement ratios of functional relevance or traditional distance measurements [1,12-16]. One important aspect of phenotypic variation in the family is the snout elongation associated with nectarivory. This elongation is thought to be responsible for a trade-off between two functional demands: tongue support and bite force [8,9,17]. Bats with longer snouts might have longer operational tongue lengths [18], but are known to have weaker bites than bats with short snouts, what might restrict the dietary range accessible to them [9] and lead to seasonal migrations [19]. Nogueira *et al*. (2009) [17] have shown that apart from the general elongation, other skull and mandible shape changes are associated with (size-independent) bite force, such as the relative size of mandibular processes (coronoid, angular), zygomatic arch position and robustness and the relative position and sizes of tooth rows. The feeding behaviour is also relevant to the understanding of the biomechanics of feeding and resource partitioning in bats [14,20], but is less studied than morphology.

The ecomorphological diversification of phyllostomids has long been considered the result of an adaptive radiation, but no specific tests of the responsible mechanisms have been performed, apart from the correlational studies mentioned before (most of them not using comparative methods). An analysis of diversification rates indicated a significant shift at the base of the phyllostomid tree [21], that could be associated with an adaptive diversification, but increased speciation is not an unequivocal evidence of adaptive radiation [22,23]. Monteiro and Nogueira (2010) [24] provide indirect evidence of adaptive evolution, assessing the integration patterns in the phyllostomid mandible during evolutionary shape changes. The interspecific integration patterns (correlated shape changes among mandibular components) were independent from pooled within-species integration patterns (which mirrored mammalian developmental genetics patterns), as expected during adaptive evolution on an adaptive landscape [25,26]. Long term selection on species means (macroevolutionary changes) is expected to be independent from the structure of genetic correlations among morphological variables, depending only on selection gradients [25,26] associated with specific adaptive peaks. In this context, we can move beyond the usual phenotype-ecology correlation approach, assessing the likelihood of different evolutionary scenarios through recent model-based approaches for comparative analyses [27,28]. It is possible to select between alternative adaptive models with different postulated selective agents or adaptive peaks [29]. It is also possible to estimate optimal phenotypic values for each adaptive peak to use as a basis for testing hypotheses regarding the adaptive evolution of lineages [29], and to cast light on the evolutionary processes responsible for species diversity [30].

We focus our study on the variation of mandible form (shape and size), which is a model for the evolution of complex morphological structures [31]. The mandible can be used as a proxy for the facial skull due to developmental integration of jaw (mandible-maxilla) parts [32]. Unlike other parts of the skull that harbour different functions (protecting the brain and sensory organs), the mandible's main functional demands are related to feeding, and dietary changes are expected to be the main selective agent for this structure. We combine ecomorphological correlations, patterns of disparity through time and a model-based comparative analysis to test evolutionary mechanisms and scenarios during the radiation of phyllostomid bats.

## Results

### Structure of morphological and dietary variation

**Figure 1:**
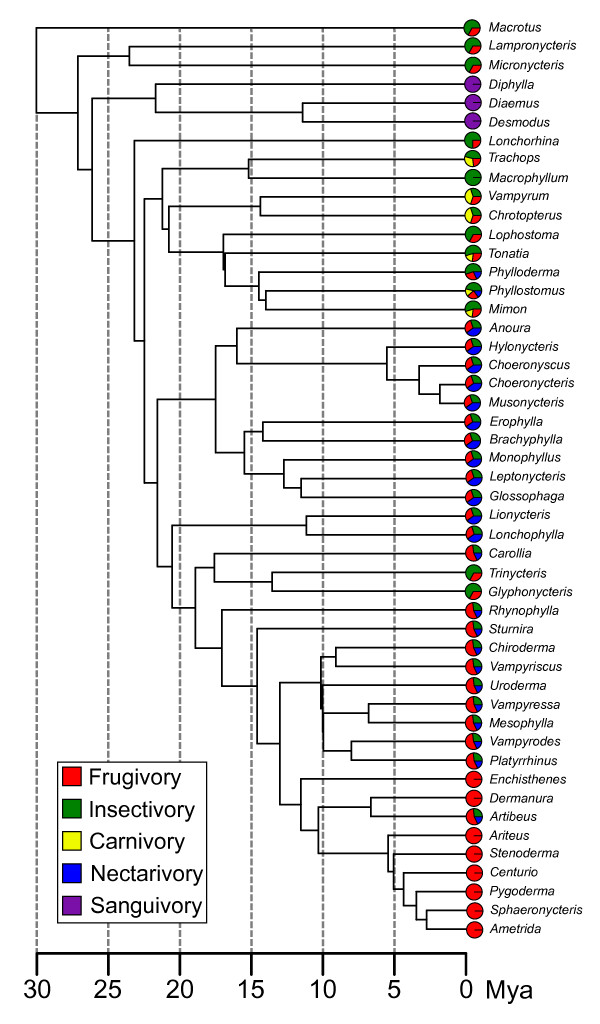
**Phylogenetic relationships among phyllostomid bats with dietary preferences**. Phylogeny based on concatenated mtDNA and RAG2 data (modified from Baker *et al*. [2003-2010] [3,33]). Branch lengths are proportional to time since divergence (in millions of years). Pie graphics depicted at tree tips depict approximate contribution of different diet items according to our ranked estimates from the literature (see methods section).

The distribution of dietary preferences along the phylogenetic tree of phyllostomids (Figure 1) indicate that most dietary shifts occurred only once during the evolution of the family, but nectarivory seems to have evolved twice independently and a return to insectivory is observed in the ancestor of *Trinycteris *and *Glyphonycteris*. Dietary specialisation has been a major theme in phyllostomid evolution, but the data from our literature review suggests that many species present mixed diets and will use both plant and animal food items, with geographical and seasonal variation in relative importance. Sanguivores and some frugivores are more strict. A principal component analysis (PCA) of dietary preferences (not shown here, but published in [24], see also the Additional file 1), shows a strong correlation between carnivory and insectivory as a feature of the first diet PC, suggesting that they might be considered a single group of animalivores. The first diet PC contrasts animalivory and frugivory as opposite trends. The summarisation of three diet variables in the first diet PC explains why there was strong multicollinearity in preliminary analyses of the diet data (see methods), justifying the PCA transformation. The second diet PC separates sanguivores from the rest and the third diet PC separates the nectarivores from the rest (the two remaining PCs were not clearly interpretable as simple diet contrasts).

**Figure 2:**
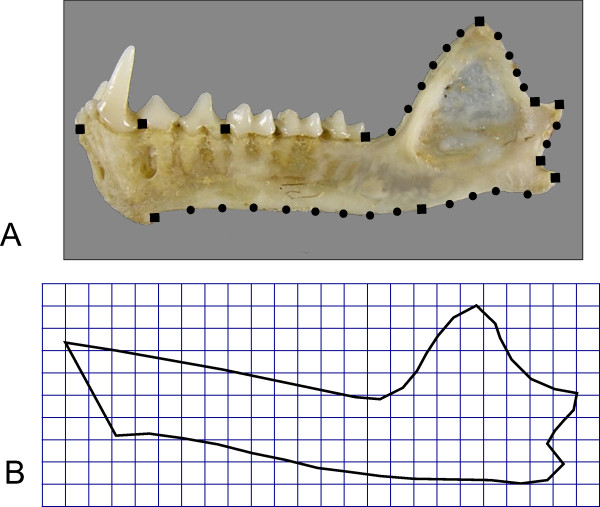
**Phyllostomid mandible showing reference points used for morphometric analysis**. (A) Mandible of *Phyllostomus hastatus*, showing landmarks (squares) and semilandmarks (circles). Landmark descriptions as in [17]. (B) Average shape calculated by Procrustes superimposition over all 49 species.

**Figure 3:**
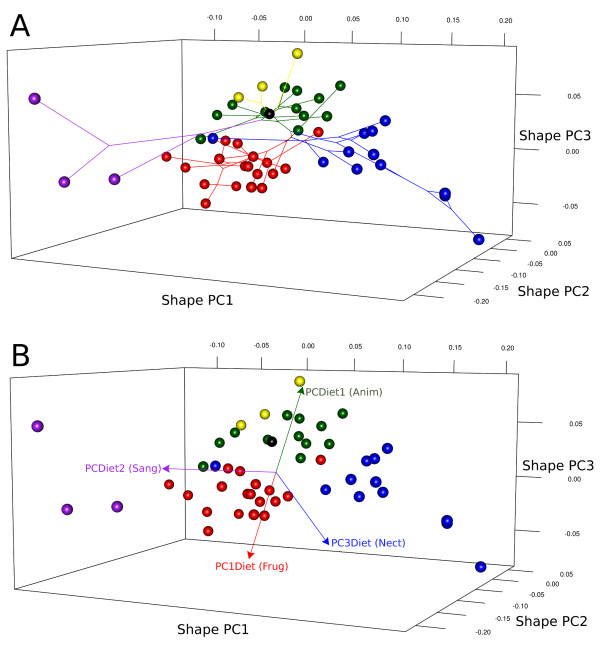
**Scatterplots of first three shape Principal Components with superimposed phylogenetic tree and diet variables**. (A) The phylogeny tips (species mean shapes) are depicted as spheres, coloured according to major dietary preferences (green - insectivores, yellow - carnivores, red - frugivores, purple - sanguivores, blue - nectarivores). Connecting lines determined by phylogeny branches, where nodes correspond to estimated ancestral shapes. The tree root shape is depicted as a black sphere. Branch colours correspond to estimated ancestral diets. (B) Scatterplot of first three shape PCs with associated diet PCs. Diet PC vectors directions based on partial correlations from the multivariate PGLS regression. Names in parentheses after diet PCs indicate the main dietary item associated with that PC direction.

The shape space for phyllostomid mandible evolution was assessed by superimposed coordinates of landmarks and semilandmarks (Figure 2). A principal component analysis (PCA) of superimposed coordinates returned five over-dispersed shape principal components, according to a parallel analysis (a Monte Carlo approach that compares observed eigenvalues with a distribution of eigenvalues from PCAs of random data sets with uncorrelated variables). The first five shape PCs explained 90.6% of total shape variation and are strongly associated with the divergence among dietary groups (Figure 3, see also Additional file 2 for an animation of the ordination with names of genera). Mapping the phylogenetic tree (with maximum likelihood ancestral character estimation) onto the space of the first three shape PCs (Figure 3A), each dietary group represents an independent direction of shape variation, with few convergences in the separate lineages (Figure 3A also depicts model OU.5 from the model-based analysis, see below). This is observed both considering the dietary groups as discrete categories (Figure 3A) and the relative contribution of diet items by multivariate regression (Figure 3B). The regression of the five shape PCs on dietary PCs and skull length (used as a more familiar and intuitive proxy for mandible centroid size - see methods) was significant at the multivariate level, but the fifth shape PC was not particularly associated with any dietary PC or skull length (Table 1). The principal component analysis of dietary variables worked satisfactorily to remove multicollinearity among independent variables, as indicated by the low variance inflation factors (≤ 1.271). It should be emphasised that the principal components of species means depict the major axes of shape change among species, without the constraint to depict shape changes associated with diet (as differences among dietary types could be aligned with none of the PCs). However, the strong association of dietary PCs with independently derived shape PCs suggests common evolutionary processes behind the ordination patterns.

**Table 1:** Multivariate PGLS regression results for the shape PCs on size and diet.

Shape PC1	*R*^2 ^= 0.570		
	*b*	*r*	*P*

CL	0.0000	0.0022	0.9886
Diet PC1	0.0303	0.2242	0.1340
Diet PC2	-0.0903	0.5631	< 0.0001
Diet PC3	0.1162	0.6312	< 0.0001

Shape PC2	*R*^2 ^= 0.807		

	*b*	*r*	*P*

CL	-0.0010	-0.2116	0.1581
Diet PC1	0.0190	0.2998	0.0429
Diet PC2	-0.1047	-0.8650	< 0.0001
Diet PC3	-0.0754	-0.7549	< 0.0001

Shape PC3	*R*^2 ^= 0.444		

	*b*	*r*	*P*

CL	-0.0008	-0.1592	0.2907
Diet PC1	0.0545	0.6420	< 0.0001
Diet PC2	0.0079	-0.1201	0.4264
Diet PC3	-0.0226	-0.3049	0.0394

Shape PC4	*R*^2 ^= 0.440		

	*b*	*r*	*P*

CL	-0.0020	-0.4482	0.0018
Diet PC1	-0.0233	-0.4031	0.0054
Diet PC2	-0.0003	-0.0056	0.9705
Diet PC3	-0.0016	-0.0273	0.8573

Shape PC5	*R*^2 ^= 0.159		

	*b*	*r*	*P*

CL	-0.0008	-0.1974	0.1884
Diet PC1	0.0151	0.2877	0.0525
Diet PC2	-0.0007	-0.0142	0.9255
Diet PC3	0.0140	0.2484	0.0960

The partial regression (*b*) and partial correlation (*r*) coefficients and their significances (*P*) are depicted for each independent variable: CL - Condylobasal Length, and diet principal components. Diet PC1 is a contrast between animalivores (positive) and frugivores (negative). Diet PC2 separates sanguivores (positive scores) from the rest. Diet PC3 separates nectarivores (positive) from the rest. The variance inflation factors were small, ranging from 1.249 (Diet PC1) to 1.007 (Diet PC2). The multivariate model was significant (Wilk's Λ = 0.0092, *F *= 15.23, *df*_1 _= 25, *df*_2 _= 150.1, *P *< 0:00001).

**Figure 4:**
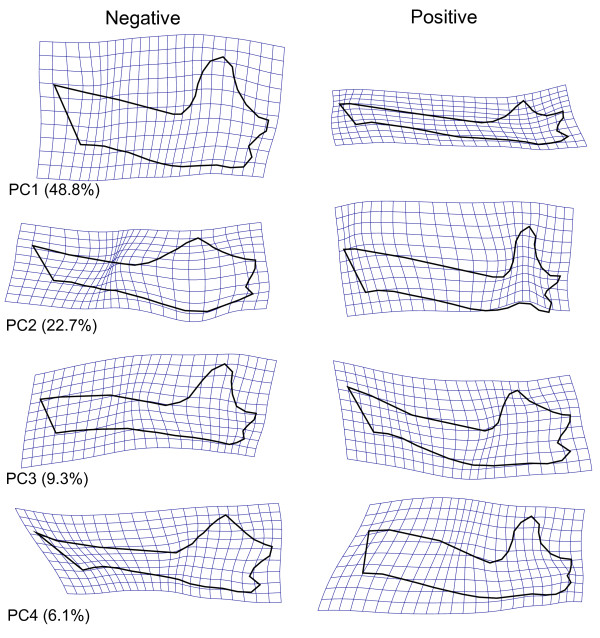
**Mandible shape variation associated with Principal Components**. Shape icons depicted as grid deformations relative to the grand average shape (Fig. 2B). Shape changes correspond to maximum observed range of scores. Numbers in parentheses indicate the amount of total shape variation explained by each PC.

The first shape vector is mostly associated with the second (sanguivory) and third (nectarivory) diet PCs in opposite directions (Figure 3B, Table 1). The patterns of shape change associated with the first shape PC are dominated by a relative lengthening (positive direction - associated with nectarivory) and shortening or deepening (negative direction - associated with sanguivory) of the mandible (Figure 4). This is not a uniform shape change, as the mandibular elongation is not evenly distributed throughout the mandible (e.g. the region below the premolars is more elongated and shortened than the region below the molars and incisors). The second shape PC is the only shape axis significantly associated with all three diet PCs (and the model with larger coefficient of determination *R*^2^). It is mostly associated with diet PCs 2 and 3 (sanguivory and nectarivory) (Table 1), but showing a shared morphological change, rather than a contrast. This axis of shape change is more closely associated with sanguivory in its negative direction (Figure 3B, Table 1), depicting a noticeable relative decrease of the molar row and a relative decrease of the coronoid process (Figure 4). This decrease in the coronoid process is also evident in nectarivores hence its significant association with diet PC3. The diets with stronger masticatory demands (frugivory and animalivory) present positive scores along shape PC2. This direction of shape change is dominated by relatively larger molar rows and larger coronoids (Figure 4). The relative increase in the molar row is a feature of animalivores, hence the weak (but significant) correlation observed between the second shape PC and the first diet PC (Table 1). The third shape PC is strongly associated with the first diet PC (Table 1), which contrasts animalivores and frugivores. The shape change depicted by this shape axis is dominated by a change in the shapes of mandibular processes and relative differences in relative sizes of molar teeth (Figure 4). In the positive direction (associated with animalivory), the coronoid is wider, the angular relatively larger and the molar teeth row longer. In the negative direction (associated with frugivory), the coronoid is narrower, slightly curved posteriorly, the angular is relatively smaller and the molar teeth row shorter (but premolars row longer). There is also a weak but significant partial correlation of the third shape PC and the third diet PC (nectarivory). The fourth shape PC explains a small, but still over-dispersed, amount of the variation among species (Figure 4). The multivariate regression results indicate that this shape feature is correlated with mean size differences (evolutionary allometry) and the first diet PC. The scatter of PC scores (not shown, but scores available in Additional file 1) ordinates apart six short-faced frugivore species (*Ametrida, Sphaeronycteris, Pygoderma, Centurio, Stenoderma, Ariteus*) comprising the clade Stenodermatina from Baker *et al*. (2003) [33]. These species are strict frugivores and present high positive scores on shape PC4. High negative scores on shape PC4 are observed among insectivores and the large carnivore bats (*Vampyrum, Chrotopterus*). The shape changes depicted by the fourth shape PC (Figure 4) are related to the mandibular processes (similar to those observed along shape PC 3) and the anterior region. A noticeable aspect of the shape change is the "chin" formed in these short-faced frugivores, as well as changes in the relative sizes of molar and premolar rows. The fifth shape PC was not associated with any dietary PC or skull length.

### Patterns of phenotypic and ecological disparity through time

**Figure 5:**
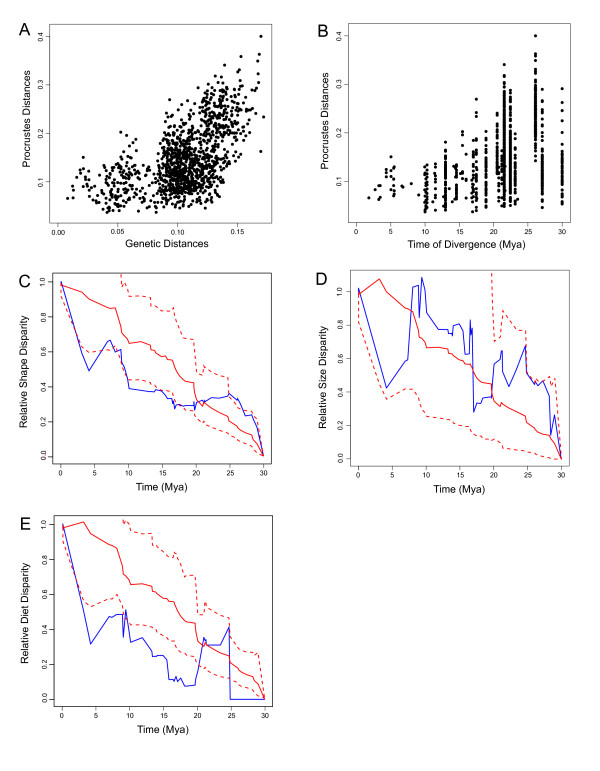
**Patterns of morphological, dietary and genetical divergence through time**. (A) Scatterplot between shape distances (Procrustes) between pairs of species and genetic distances (percent sequence divergence [33]) for the same pairs. (B) Scatterplot between shape distances (Procrustes) between pairs of species and time since divergence. (C) Line plot showing changes in relative morphological disparity through time (DTT). Relative morphological disparity measured as average squared Euclidean distances (with the full set of shape PCs) among existing clades at a given point in relative time. The blue solid line shows the observed disparity, and the red dashed line shows the expected disparity under a neutral evolution model as the average of 1000 simulations. (D) Same as C, but showing changes in relative size disparity through time. (E) Same as C, but showing changes in relative dietary disparity through time. Relative dietary disparity measured as average Manhattan distances using relative importance of diet items for each species.

The scatterplot of morphometric (Procrustes) distances versus genetic distances shows a combination of two scatter patterns (Figure 5A). For larger genetic distances (> 0.10), there is a positive linear relationship between the two types of distances, whereas for small genetic distances (< 0.10) there seems to be no relationship. This distance plot suggests at least two different evolutionary processes operating at different moments along the evolution of phyllostomids. The inference of evolutionary modes can be made qualitatively by comparison with Polly's simulated patterns of morphological divergence (see Figure 16 in Polly (2004) [34]). The linear relationship between distances with a steep slope is expected under directional selection, and the lack of relationship is expected under stabilising selection. The scatterplot comparing morphometric distances and time since divergence (Figure 5B) shows a less clear pattern at large times since divergence due to species that are phenotypically and (to a certain extent) genetically similar but diverged early (27-30 Mya) during phyllostomid evolution (*Macrotus, Lampronycteris, Micronycteris *and the other insectivores - see Figure 1). This pattern suggests that the early period of phyllostomid divergence was marked by a combination of directional and stabilising selection.

The disparity through time (DTT) plots provide further evidence of a two-mode evolutionary history for shape and diet (Figures 5C and 5E). These plots show average sums of morphometric and dietary distances (relative to the total sum or total disparity) for lineages of a given (relative) age range through the phylogenetic tree. The early evolution of phyllostomid lineages is marked by larger disparity (blue solid line) than expected by 1000 neutral evolution simulations (median and 95% confidence intervals of simulations depicted as solid and dashed red lines, respectively). During this initial period, there is a high average disparity within existing lineages. Both shape and diet disparities peak above the expected neutral disparity around 25 Mya. There is also a turning point after this early diversification (around 20 Mya), when observed disparity within lineages is smaller than expected under neutral evolution, suggesting that stabilising selection was the main evolutionary force constraining diversification within lineages after the main ecological divergence had taken place. Size disparity through time follows a similar pattern initially, but there is a second peak above expected median disparity between 16-7 Mya. This second peak is unique for size disparity and takes place after the main dietary categories were established. The relevance of this pattern is not so clear because the confidence limits for univariate characters are considerably large and much of the size disparity fluctuation within lineages falls inside the confidence intervals. The five panels (Figure 5A, B, C, D, E) are complementary in the sense that they are indicating a duality of evolutionary processes at different times during the phyllostomid radiation, and that shape and size did not have a correlated pattern of disparity through time. The steep slopes observed in the right hand extremity of Figures 5A and 5B are consistent with the larger than expected shape disparity in the deeper parts of the tree (between 30 and 20 Mya - Figure 5C), and both are predicted under directional selection. The near zero slopes in the left hand extremity of Figures 5A and 5B are consistent with the lower than expected disparity within younger clades (Figures 5C and 5D), and both patterns are likely under stabilising selection.

### Evolutionary models of phenotypic divergence

**Figure 6:**
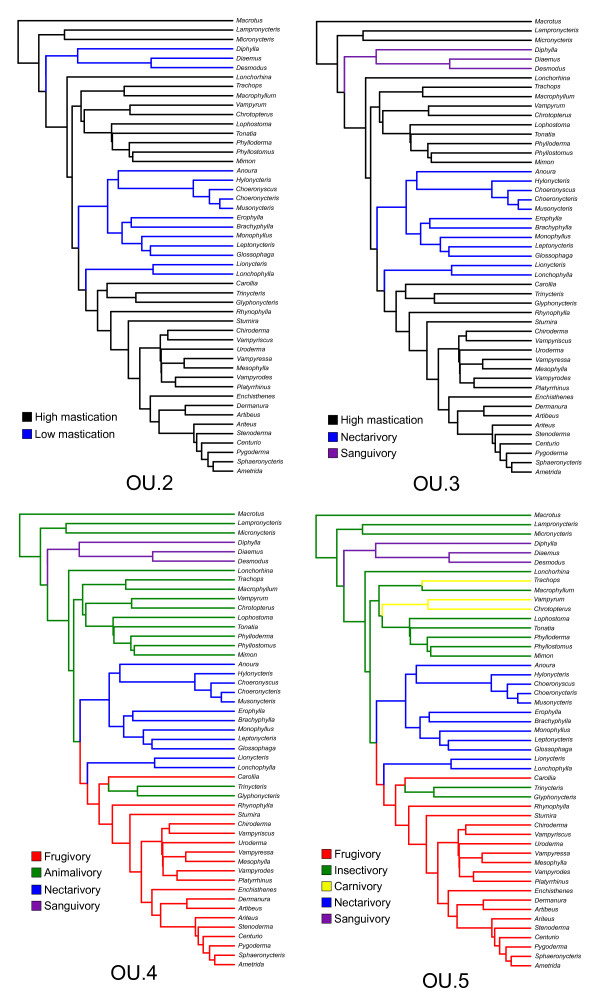
**Adaptive regime models for association between mandible shape and diet**. Model OU.2 discriminates two adaptive optima, one for species with feeding modes that involve a considerable amount of mastication (Insectivory, Carnivory and Frugivory), and species with feeding modes involving little or no mastication (Sanguivory and Nectarivory). Model OU.3 discriminates three adaptive optima: two with little or no mastication, but with fundamental differences (Sanguivory and Nectarivory), and one involving mastication (lumping Insectivory, Carnivory and Frugivory). Model OU.4 discriminates four dietary adaptive optima, lumping Insectivores and Carnivores in the Animalivore category. Model OU.5 discriminates optima for the five main dietary groups. All ancestral states estimated by a maximum likelihood algorithm (see methods).

We tested five alternative models of the evolution of mandible shape (Figure 6, Table 2). The first model (BM) was a neutral Brownian motion along the phyllostomid phylogenetic tree, whereas the remaining models (OU.2-OU.5) incorporated a deterministic component (selection) similar to adaptive radiations with varying numbers of fitness peaks (2 to 5), according to increasingly complex ecological assumptions, causing dietary types to be lumped together or split into separate peaks. The model-fitting results are summarised in Tables 2 and 3. The phenotypic variables used were the first three or five shape PCs (multivariate models were used for fitting all PCs at the same time). Fitting multivariate models with different numbers of variables allowed for some insight into the sensitivity of results to model degrees of freedom. Using more shape variables increases the amount of information about variation patterns in the model, but also increases the number of parameters, causing problems for estimation. The criteria used for model selection: weights of Akaike information criteria corrected for sample size w(AICc) and weights of Schwarz information criteria w(SIC) for a particular model *M_i _*can be interpreted as the probability of *M_i _*being the "best" model given the data and the set of candidate models (the SIC being more conservative in relation to model complexity than AICc). For the models with three PCs, according to all criteria used, the best fitting model was OU.4, an Ornstein-Uhlenbeck process (stabilising selection) with four adaptive peaks (frugivores, nectarivores, sanguivores and animalivores). For the models with five shape PCs, however, the increased complexity of the models (as measured by the number of parameters being estimated (the degrees of freedom in Table 2) caused the model OU.5 (the most complex model) to have the higher w(AICc), but the Brownian motion (the simplest model) to have the higher w(SIC). This is an expected result of model dimensionality, since the SIC criterion penalises more complex models more severely than the AICc.

**Table 2:** Performance of alternative models for the evolution of mandible shape using principal components.

Models with 3 PCs					
	BM	OU.2	OU.3	OU.4	OU.5

AICc	-503.15	-521.09	-557.23	-571.23	-568.09
Δ(AICc)	68.08	50.14	14	0	3.14
W(AICc)	0.0000	0.0000	0.0008	0.8272	0.1721
SIC	-477.55	-472.61	-501.83	-509.29	-500.05
Δ(SIC)	31.74	36.68	7.46	0	9.24
w(SIC)	0.0000	0.0000	0.0232	0.9673	0.0095
DOF	9	18	21	24	27

Models with 5 PCs					

	BM	OU.2	OU.3	OU.4	OU.5

AICc	-953.13	-963.13	-994.16	-1016.73	-1022.49
Δ(AICc)	69.36	59.36	28.33	5.76	0
w(AICc)	0.0000	0.0000	0.0000	0.0532	0.9468
SIC	-886.85	-839.13	-857.41	-867.95	-862.51
Δ(SIC)	0	47.72	29.44	18.9	24.34
W(SIC)	0.9999	0.0000	0.0000	0.0001	0.0000
DOF	20	40	45	50	55

**Figure 7:**
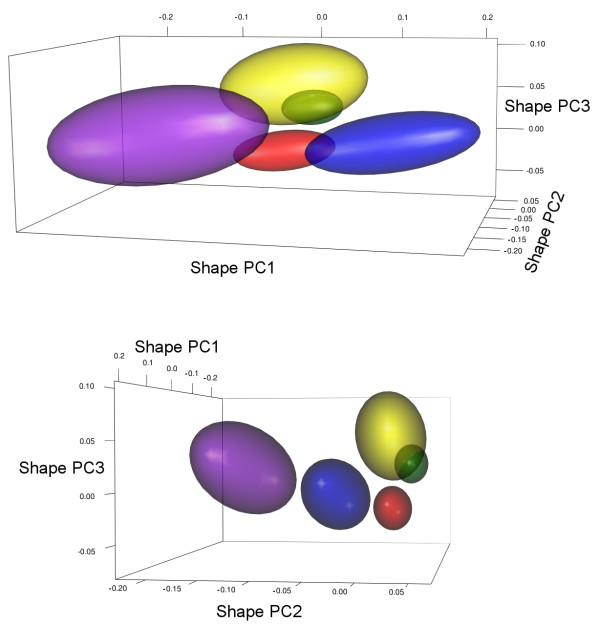
**Bootstrap confidence ellipsoids for dietary adaptive optima (*θ_i_*) in shape space**. Confidence ellipsoids (95%) calculated after bootstrap resampling under model OU.5 for five shape PCs (only the first three PCs shown). Dietary optima ellipsoids are colour-coded following key in Figs. 3 and 6 (OU.5).

Model names defined as follows: BM - Brownian motion, OU - Ornstein-Uhlenbeck with two (OU.2), three (OU.3), four (OU.4), and five (OU.5) adaptive optima (see text and Fig. 7 for details). AICc is the corrected Akaike Information Criterion for small sample sizes, Δ(AICc) is the difference relative to the model with smaller AICc, w(AICc) is the Akaike weight for each model. SIC is the Schwartz Information Criterion, Δ(SIC) and w(SIC) are the differences and weights as defined above. DOF are the numbers of parameters estimated by each model. All models were multivariate. The upper part of the table shows models fitting the first three shape PCs simultaneously, whereas the bottom part of the table shows models fitting the first five shape PCs.

**Table 3:** Performance of alternative models for the evolution of skull length.

	BM	OU.2	OU.3	OU.4	OU.5
AICc	307.59	309.30	311.75	311.86	298.11
Δ(AICc)	9.48	11.19	13.64	13.74	0
w(AICc)	0.0086	0.0037	0.0011	0.0010	0.9856
SIC	311.11	315.96	319.82	321.21	308.62
Δ(SIC)	2.49	7.33	11.19	12.58	0
w(SIC)	0.2184	0.0194	0.0028	0.0014	0.7580
DOF	2	4	5	6	7

Model names defined as follows: BM - Brownian motion, OU - Ornstein-Uhlenbeck with two (OU.2), three (OU.3), four (OU.4), and five (OU.5) adaptive optima (see text and Fig. 7 for details). AICc is the corrected Akaike Information Criterion for small sample sizes, Δ(AICc) is the difference relative to the model with smaller AICc, w(AICc) is the Akaike weight for each model. SIC is the Schwartz Information Criterion, Δ(SIC) and w(SIC) are the differences and weights as defined above. DOF are the numbers of parameters estimated by each model.

**Figure 8:**
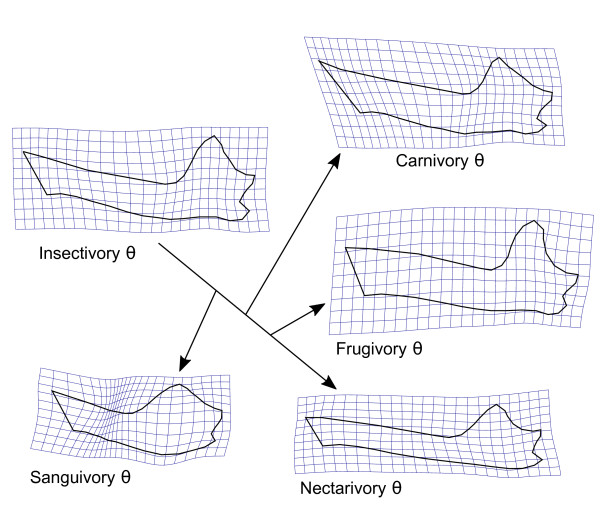
**Shape changes associated with dietary adaptive optima (*θ_i_*) for the best fitting evolutionary model**. Shape icons for each diet optimum depicted as grid deformations relative to the grand average shape (Fig. 2B). Shape changes obtained by shape principal component scores estimated as *θ_i _*by the model OU.5 (Fig. 6). Arrows indicate directions of evolutionary change between adaptive peaks according to ancestral diet estimates.

The 95% confidence ellipsoids for predicted optimal shapes (*θ_i_*) from model OU.5 do not overlap in the space spanned by the first five shape PCs (Figure 7, but only the first three PCs are shown). Their centroid positions correspond roughly to the averages of points with the same colours in Figure 3. There is some overlap between carnivore and insectivore ellipsoids (about half the insectivore ellipsoid is within the carnivore ellipsoid), and the complete separation of the two peaks is only achieved in the scores of the fifth shape PC (result not shown). This is consistent with the numeric results in Table 2 where the "best" model with three PCs lumps carnivores and insectivores into an animalivore peak. The predicted optimal shapes for each dietary adaptive peak in the best fitting model OU.5 (Figure 8) reproduce the shape changes described above in the correlational analysis. The grand average shape (Figure 2B) was used as reference for the grid deformations, but the patterns of shape changes are the same if using an estimated root shape for the phyllostomid tree (using MorphoJ to map shape onto phylogeny [35]), as the difference between the grand average and the estimated root shape are negligible (results not shown). The earliest adaptive peak is the one for insectivores, with a relatively large molar series and well developed mandibular processes. The first off shoot to an alternative adaptive peak is towards sanguivory, where the molar series is greatly reduced and the mandibular processes are reduced (but the central part of the ramus is relatively expanded). The carnivory optimum shape shares the enlarged molar series with insectivores, but the mandible is deeper. Further changes occurred independently towards frugivory and nectarivory. The optimal shape for frugivores present a higher mandible with a smaller angle at the symphysis. The mandibular processes are relatively well developed and the posterior border of the coronoid forms a characteristic curve (the coronoid of animalivores has a straight border). The shift to the nectarivore adaptive peak involved an elongation of the mandible and a corresponding decrease of mandibular processes, particularly the coronoid.

**Figure 9:**
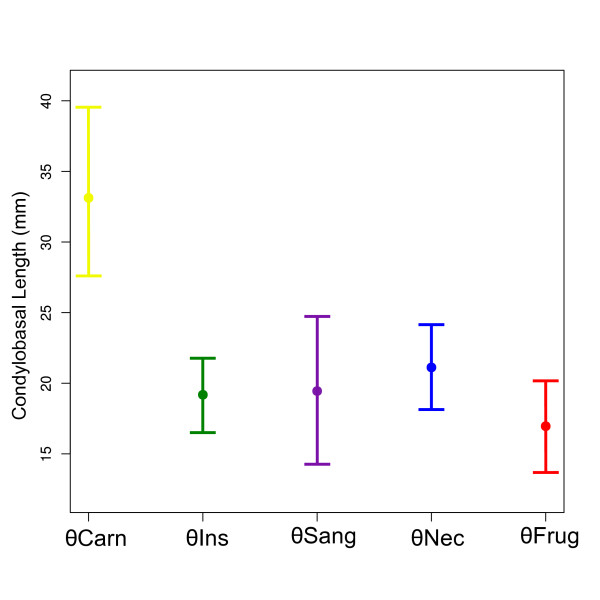
**Cranial size adaptive optima (*θ_i_*) for the best fitting evolutionary model**. Size optima (Condylobasal Length) and confidence intervals for each dietary adaptive peak in model OU.5. Circles represent median and whiskers represent 95% confidence intervals of bootstrap resampling estimates for each *θ*.

We also tested five alternative models for the evolution of skull length (Figure 6, Table 3). According to both AICc and SIC weights, the best fitting model was OU.5, an Ornstein-Uhlenbeck process with five adaptive peaks (one for each main dietary category considered). In fact, OU.5 was the only model that performed significantly better than Brownian motion. The five size optima (*θ_i_*) estimates and their 95% confidence intervals (Figure 9) show a considerable overlap with the exception of the optimum for carnivory, what explains the best fit presented by OU.5. It is the only model that separates carnivores from insectivores. Frugivores and nectarivores present smaller and larger skull length optima relative to insectivores, but these estimates fall within the confidence interval for insectivory. When interpreting these results, it is important to bear in mind that skull length is not a proxy for body mass and will be influenced by patterns of cranial elongation or shortening.

## Discussion

The combination of ecomorphological correlations, disparity analysis and model-based comparative techniques provided strong evidence for the evolutionary mechanisms responsible for the phyllostomid radiation. Although there are a few instances of convergence in phyllostomids [3,4], the dietary divergence presents a strong phylogenetic structure [24,36], where most specialisations occurred only once and many of the main lineages (recognised as subfamilies in most taxonomic studies [33]) are relatively homogeneous morphologically and ecologically. Convergence is an important part of the study of adaptation [37], and its absence makes it harder to infer the role of natural selection in biological diversification. One has to be particularly careful with the comparative methods and evolutionary model assumptions, for using the wrong models or methods are likely to lead to erroneous conclusions [28,38,39]. For example, in the present study, forcing a Brownian model of evolution into the phylogenetic regression model would lead to non-significant associations among shape and diet principal components (results not shown). Flexible approaches allowing for different evolutionary models [27,28,40] provide more sophisticated and interpretable results [39], as well as more interesting questions to be explored. The model-based approach used in this study shed light on the evolutionary mechanisms responsible for the evolutionary radiation of phyllostomid bats and makes it possible to generate predictions of optimal shapes (or ratios of functional relevance) for dietary specialisations that can be used in the future to test hypotheses of biomechanical optimisation [41].

The major axis of shape variation among phyllostomid species (first shape principal component) ordinates species with contrasting degrees of rostral elongation. This shape change is regarded by Freeman (2000) [1] as one of the "cheap tricks" of mammal diversification, and studies on other groups of mammals such as domestic dogs [42] suggest that this pattern of shape variation can arise in a microevolutionary scale (~10000 years) as a response to selective processes. Rostral elongation in phyllostomids is associated with a trade-off between support for the elongated tongue and bite force (species with shorter rostra usually have stronger bites) [17,43,44]. This reduced bite force is a result not only of the increased out-lever [44], but also of the relative decrease of muscle insertion areas and robustness of elongated skulls and mandibles [17]. The bite force constraint caused by morphological specialisation is known to limit the dietary scope [8,9] and might have important ecological consequences, particularly regarding patterns of resource use and foraging strategies within guilds [19].

Mandible and palate length are generally good predictors of operational tongue length in most nectarivores [18] (but see [45,46] for an alternative adaptation in *Anoura fistulata*). Longer tongues are considered an adaptation for nectar extraction, not only because these will reach flowers with longer corollas [46], but because they allow the nectar specialists to explore a larger variety of plant species more efficiently (avoiding seasonal changes to insects and fruit when local nectar abundance decreases) [19,47], and possibly to maintain a longer distance from the flower during feeding (reducing predation risk) [18]. It is currently established that nectarivory has evolved twice independently within phyllostomids [3,4], and despite a superficial phenotypic convergence (both lineages do present the mandibular elongation), there are many anatomical differences, particularly in tongue morphology [4]. All this morphological variation suggests that, in this system, there are many ways to achieve the same function (many-to-one relationships [48]) and that there is more variation in diet and foraging strategies among nectarivores than implied by the common use of such category. *Brachyphylla *is a singular nectarivore in the sense that it presents a short mandible (the blue sphere with negative score on shape PC1 - Figure 3A, see also Additional file 2) and is phenotypically similar to *Phylloderma *and *Sturnira*, being ordinated between frugivore and insectivore species. The similarity between *Brachyphylla *and the frugivore Stenodermatine lineage has been recognised for other morphological characters in the dentition and cranial shape [1,49] and could be the result of dietary changes to exploit open niches during island colonisation, as suggested by Griffiths (1985) [49].

The second principal component of interspecific shape variation depicts a common pattern of shape change for diets with low mastication (shared features between nectarivores and sanguivores) versus high mastication (shared features between animalivores and frugivores). Both sanguivore and nectarivore specialists do present lower bite forces than expected for their sizes [8]. Although nectarivores and sanguivores are in extreme opposites regarding the mandibular elongation pattern of shape PC1, they do present similarities in the relative reduction of mandibular processes (particularly the coronoid) and molar series (more pronounced in sanguivores). These shared features are consistent with developmental consequences of selection for morphological specialisations [24]. This is clear for nectarivores, where selection for a longer, narrower skull has limited the relative size of muscles and their areas of attachment [17]. The explanation for the pattern of shape changes in sanguivores is not as direct as that for nectarivores, as the evolution of different mandibular components towards this adaptive peak has been shown to disagree with expected developmental patterns [24], where tooth bearing components and mandibular processes form almost independent modules. This is because the two tooth bearing components seem to change their shapes independently: a relative increase in the anterior alveolar (incisives and canines), combined with a relative decrease in the posterior alveolar (molar series). This emphasis in anterior dentition is also observed in tooth development as well, and was hypothesised to be the result of selection to sanguivory [50]. Therefore, even though a reduction of bite force and muscle masses is observed in both sanguivores and nectarivores [8,51], the developmental consequences of these changes are more localised (in the posterior mandible) in sanguivores than in nectarivores. This is probably due to stronger stabilising selection in the anterior region of the mandible of sanguivores [24]. The evolution of sanguivory has been discussed in the literature, but the current limitations of available data make it difficult to get past the delineation of general scenarios and hypotheses [3] to actually testing functional predictions about agents of selection and phenotypic variation. Sanguivore skulls did not evolve according to expected developmental integration patterns [24], and do not meet traditional biomechanical predictions based on models of mastication [16]. The functional demands on the skull associated with sanguivory are not known in detail, but should include not only biting off flesh and lapping blood, but also sensorial tasks in finding and approaching potential bloodmeal sources. Sanguivores are unique among phyllostomids in that there are no known intermediate forms (both in diet and phenotype), whereas for the species that specialised in plant items (frugivores and nectarivores) there are plenty of intermediate species with mixed diets. This pattern suggests that the divergence of frugivores and nectarivores might have occurred along ridges and local maxima on the adaptive landscape, but the evolution of sanguivores has probably occurred via a leap over a fitness valley in a short period of time (between 31 and 21 Mya [3]). Because the transition period was very short, useful fossils documenting it would need to be obtained from this specific window on the late Oligocene. However, all known fossils of vampire bats are Plio-Pleistocenic *Desmodus *and *Diphylla *[52], which are the same or very similar to extant vampire species.

The first and second major axes of variation in the mandibular shape space of phyllostomids mostly contrast groups that present high and low masticatory demands. The phenotypic differences between dietary specialists with high masticatory demands (frugivores and animalivores) are depicted only on the third and fourth principal components of shape. These differences are associated with relative positions and sizes of mandibular processes (lower condyle, smaller angular in frugivores), tooth row lengths (longer molar rows in animalivores), and the anterior region of the mandible (mandible shorter and deeper, forming a "chin" in frugivores). Aside from large beetles (which seem to be harder than any other food item processed by bats [9]), insects and fruit do not present significantly different hardnesses and there is a lot of variation within these general groups, mostly due to size differences of the dietary items (larger items are harder [9,15,53]). Animalivores and frugivores do not present noticeable differences in size-independent bite forces [9,17], and there is a considerable amount of behavioural plasticity that allows them to modulate the bite force according to food hardness [20]. The extensive shape changes in the skull, mandible and dentition [1,13] observed in the evolutionary transition towards frugivory did not seem to be associated with changes in bite force [17]. Different from sanguivores and nectarivores, frugivores needed to maintain the bite strength for mastication, while adjusting for their specific functional needs (which are varied, according to the main type of fruit consumed [6,13,15,54]). The morphological changes observed in frugivores seem to sacrifice precision of dental occlusion (a feature apparently more important in animalivores) to favour skull and mandible robustness [13], while maintaining a small body size [15], particularly in the shorter, deeper mandibles observed in the small clade of specialist frugivores (the Stenodermatina).

Allometric variation is a controversial theme in phyllostomid evolution and ecology. There is no doubt that body size is the main factor causing differences of absolute bite force [8] and will have a consequence on the dietary scope of particular species [9,12]. On the other hand, small frugivores, such as *Centurio *are able to produce higher bite forces than expected for their sizes [43] due to cranial shape changes. Carnivory is traditionally associated with an increase in body size and is considered an allometric extrapolation of insectivory [36]. One small group of strict frugivores (Stenodermatina, see above) presents small average size, but it is not clear whether their size evolution was associated with diet or with the postulated insular origin of the clade [55] (see [56] for a review of insular size patterns in vertebrates). No obvious size trends can be associated with nectarivores or sanguivores. The ecological evidence for size as a structuring influence in phyllostomid communities is equivocal [57], even when considering more detailed dietary compositions. Our results do show that the evolutionary trajectories followed by diverging phyllostomid lineages in mandible shape space were too complex to be explained by simple allometric changes, and the shape changes associated with size differences, as measured by skull length (towards extreme frugivores and carnivores) were small, when compared to the drastic shape changes associated with nectarivory and sanguivory, which did not involve significant size changes. Shape and size divergence patterns seem to be decoupled in phyllostomid evolution (see also discussion below), but size is nevertheless a relevant dimension in the phyllostomid adaptive landscape. A measure of body size would probably be more suited for such ecological associations than cranial length.

The patterns of morphological and ecological divergence over time were informative regarding possible evolutionary mechanisms in the radiation of phyllostomid bats. When considering the parallel evolution of mandibular shape and diet, one sees clearly a two step radiation, marked by strong directional selection and divergence in the first phase (30-20 Mya) followed by stabilising selection and stasis in the second phase, after the main phyllostomid lineages (and diet specialities) appeared. The scatterplots comparing morphometric distances with genetic distances and time since divergence can be qualitatively compared to patterns from Polly's simulations [34] of evolutionary mechanisms. Different mechanisms are expected to leave distinctive imprints in the patterns of morphological divergence over time. In real data, noisy patterns are expected from the juxtaposition or superimposition of different evolutionary processes and it is harder to extricate the processes from the patterns. The scatterplots of genetic distances (percentage of sequence divergence), time since divergence and morphometric distances in Figures 5A, B show a two-phase pattern, which is more obvious in Figure 5A (genetic distances) than in Figure 5B (time since divergence) because of species that are phenotypically and genetically similar (insectivores) but have diverged in beginning of the radiation. The disparity through time plots for mandible shape and diet suggest also a two-phased radiation, marked initially by larger disparity than expected by neutral evolution simulations and changing lo lower disparity than expected by neutral evolution after around 20 Mya (the transition between Oligocene and Miocene - see discussion of the time frame for the phyllostomid diversification in [3,4]). At this transition point, the main lineages leading to dietary specialisations were established and most shape and diet disparity is observed among these lineages. Size disparity presents a different pattern from shape and diet, with at least two distinctive peaks at different times in phyllostomid evolution. The first peak occurring between 30 and 20 Mya, and it was probably caused by the large carnivores sharing the same branch with other groups until nearly 20 Mya (these are very different in skull length but not so different in mandible shape). The second peak happens between 16-7 Mya and is likely to be caused by disparity within Stenodermatinae [33] (The clade in Figure 1 joining species from *Sturnira *to *Ametrida*). The Stenodermatina (the clade joining species from *Ariteus *to *Ametrida*) have the shorter skulls among the phyllostomids (all of them smaller than 14 mm average - see Additional file 1), the within lineage disparity only decreases after they separate from the other Stenodermatinae (which have skull lengths comparable to the remaining lineages) around 10 Mya. Because the confidence limits for the neutral simulations are very wide, the fluctuation of size disparity through time never exceeds the expected disparity. On the other hand, it is not clear whether these confidence limits are too conservative or realistic. Therefore we have interpreted and discussed the size disparity results. This pattern of size disparity is clearly not associated with the mandibular shape disparity and dietary disparity. From the other analyses performed in this study, it is clear that evolutionary allometry is a significant but minor influence in mandibular shape diversification. On the other hand, the lack of correspondence between diet disparity and size disparity might also be explained by the lack of detail of the diet data within major categories.

The radiation of phyllostomid bats has commonly been referred to as an adaptive radiation [1,16]. However, a complete test of this hypothesis has not been thoroughly performed (although different criteria have been examined separately). According to Schluter (2000) [22], there are four criteria to determine if a radiation was adaptive: common ancestry, phenotype-environment correlation, trait utility and rapid speciation. The monophyly of phyllostomids (common ancestry criterion) was long established by a number of studies using molecular and morphological data sets [3-5,21,33]. The rapid speciation criterion has been assessed by Jones et al [21] whose findings indicate a statistically significant shift in diversification rates in phyllostomids (a hypothesis that would not be testable with the incomplete tree used in the present study). These authors found two significant shifts in phyllostomids: one just after the vampire bats (Desmodontinae) branch out and one within the genus *Artibeus*. The first diversification rate shift observed by Jones et al [21] within phyllostomids coincides with the morphological and ecological disparity peak observed by us at the base of the phylogenetic tree. The phenotype-environment correlation and trait utility criteria have been discussed before in the literature and in the present study. The radiation of phyllostomids in mandibular shape space occurred along axes leading to postulated adaptive peaks determined by the relative importance of dietary items. The shape changes along these main axes of variation have a clear functional relevance for the acquisition and processing of food, as discussed above and in the literature [1,16,17,24,58].

The model-based analysis presented in this study provided quantitative evidence of evolutionary mechanisms and scenarios. The models describing phenotypic evolution on an adaptive landscape with five different peaks fit the data better than the neutral evolution model (Brownian motion) and simpler models with less peaks. The favoured scenario combining all analyses would be one early burst of phenotypic and ecological diversification caused by directional selection towards five different adaptive peaks (insectivory, carnivory, sanguivory, nectarivory and frugivory). In our interpretations, we gave more weight to the Akaike information criteria corrected for sample size because in simulations, it performs better than the Schwarz information criteria when reality is assumed to be infinitely dimensional and the true model is not in the candidate set [59]. The results for models with different numbers of variables (shape PCs) show how sensitive the methods are to the number of parameters being estimated. In the models with the first three shape PCs, the number of parameters being estimated (DOF in Table 2) is never larger than the number of species (49), whereas with the first five shape PCs, OU.4 and OU.5 (the best fitting models otherwise) do exceed the number of species and are severely penalised by the more conservative criterion (SIC, which assumes that the true model is in the candidate set and is low dimensional [59]). As a consequence, Brownian motion is suggested as the best fitting model because it requires estimation of the smallest number of parameters. These results indicate that we were working at the limit of model complexity given our sample size, and more complex models would require considerably larger data sets (in terms of number of species) to avoid problems in estimation and model comparisons.

When using the first three shape PCs, model OU.4 was favoured, whereas model OU.5 was favoured by the models with five PCs. The separation of the carnivore from the insectivore peak is only accomplished when the fifth PC is added to the models. One might expect that five groups would span a space with four dimensions, however, because principal components do not maximise among group differences, there will be processes other than diet-related adaptive changes contributing to the observed interspecific variation patterns. These could be associated with neutral evolution or changes related to factors not measured in this study. In any case, it is a noticeable point that Horn's parallel analysis used to choose the number of principal components to be considered in the comparative analyses, independently indicated the number of components needed to discern all possible adaptive peaks in the most complex model. Parallel analysis is often shown by statistical simulations to be the best performing method to choose principal components [60,61].

The early burst of phyllostomid divergence was followed by consistent stabilising selection keeping mandible shape relatively constant around postulated dietary optima. The apparent phenotypic and ecological stasis that persists from the early Miocene (20 Mya) to the present was not followed by a lack of speciation, and the lineages of specialist nectarivores and frugivores seem to be particularly speciose [4]. This pattern suggests either a non-adaptive radiation [22] or agents of selection not specifically examined in the present study. Allopatry and biogeographic distribution patterns account for a considerable proportion of speciation within diverse phyllostomid genera where stabilising selection seems to constrain phenotypic and ecological variation [62-64]. In fact, niche conservatism and stabilising selection are expected to play a significant role in allopatric speciation processes [65], what seems to fit well with the environmental changes during the Tertiary [4,66], when the global temperature decrease, tectonic processes (Andes uplift) and sea level fluctuations created large expanses of dry open areas in South America and are linked with the diversification of many other mammal clades [67-69]. On the other hand, further selective episodes cannot be discarded, as there might be smaller adaptive peaks within the main dietary groups and the temporal and spatial variation in resource availability (particularly fruits and flowers) might generate randomly fluctuating selection that is hardly discernible from a Brownian motion [25,34]. Frugivore phyllostomid species are known to largely occur in sympatry [10,15], possibly due to further specialisation, such as the dichotomy between ground-story frugivores and fig-feeders [15], or the recently discovered granivory [6] associated with functional and morphological specialisations [58]. Nectarivore guilds can also be diverse [19] due to nomadic behaviour, seasonal and spatial changes in resource use [18,47] and different degrees of specialisation and dietary item mixtures [70]. The radiation of phyllostomid bats is actually a number of radiation episodes nested within each other, caused by a mixture of adaptive and non-adaptive evolutionary mechanisms. This is expected as real data is more likely to fall along a continuum caused by a mixture of evolutionary processes, rather than fit yes/no definitions for the adaptive radiation metaphor [71].

The radiation of phyllostomid bats is a unique example among mammals in terms of ecological and phenotypic diversity, and should be considered an important model system for studies of the evolution of functional optimisation due to the ecological diversity under the constraints imposed by powered flight [72]. Further research should provide a deeper understanding of the myriad of evolutionary mechanisms at work in this lineage. The foundations of phyllostomid ecomorphology, based on rather limited morphological, ecological, phylogenetic and functional data painted an interesting picture and provided important references and new questions to be addressed. Recent contributions comprise functional studies [41,73] that go beyond traditional biomechanics to examine the functional consequences of shape change in terms of energy efficiency and structural resistance, comparative analyses of detailed morphological, functional, and ecological data [17,24], comparative analyses combining field data on function, behaviour and ecology [7-9,20,43,44,74] and phylogenetic diversification patterns using complete species-level trees [21]. Whereas a lot of attention has been paid to the biomechanics of mastication and bite force, the functional demands associated with nectar and blood-feeding are still underrepresented in the literature. Of particular importance would be the identification of functionally relevant measurements (with fitness consequences) with regard to different dietary compositions to be associated with phenotypic data. When comparing the abundance of phyllostomid species in scientific collections, Freeman (2000) [1] has pointed out that species with specialised phenotypes and diets are rarer than the species with intermediate phenotypes and mixed diets. Are dietary specialisations equivalent to local optima on the adaptive landscape with lower fitness than optima for mixed diets? This is an important question for future research with possible implications for the dynamics of assemblages [7] and biodiversity conservation. Other promising areas are the ecological influences on cranial phenotypes outside dietary differences, such as echolocation and roost building [1,75]. The ever increasing literature is producing a massive database of morphological, ecological, functional and phylogenetic data that will be instrumental to elucidate the questions in future studies of phyllostomid ecomorphology.

## Conclusions

The patterns of phenotypic and ecological divergence are consistent with an evolutionary scenario with at least five main adaptive peaks during the early radiation of phyllostomid bats. Starting from an insectivore ancestor around 30 Mya, different lineages specialised into carnivores, sanguivores, frugivores and nectarivores. Some species are strict diet specialists with extreme morphologies, but a considerable number present intermediate diets and phenotypes, possibly as a result of adaptive ridges or multiple local optima (many phenotypes with equivalent or similar fitness) and geographical/temporal variation in resource availability. Nevertheless, some trade-offs are clear, such as the mandibular elongation observed in specialised nectarivores, which supports a longer tongue but decreases bite force changing the scope of usable resources (increased variety of flowers versus harder fruit and insects). Size and shape have evolved almost independently in this family, where the major axes of shape change (towards nectarivory and sanguivory) in phyllostomid mandibular morphospace were not correlated with size differences. On the other hand, the most impressive size differences (towards carnivory) are associated with minor shape changes. After the early burst of ecomorphological divergence, when directional selection was possibly a dominating evolutionary mechanism, some of the lineages went into a long period of stasis or non-adaptive radiations, but further selective episodes cannot be ruled out, particularly for size variation among frugivores, although the role of diet as selection agent is not entirely clear in this case. The radiation of phyllostomid bats was marked by a complex mixture of adaptive and non-adaptive mechanisms through a period of extreme environmental changes when new ecological niches were probably emerging and disappearing quickly. This led the leaf-nosed bats to present unparalleled morphological and ecological variation among mammals, which, together with the vast amount of information available in the literature, makes phyllostomids one of the most important model systems for the study of morphological, functional and ecological diversification.

## Methods

### Samples, diet and morphological data

A total of 443 specimens representative of 49 genera of phyllostomid bats were analysed (Figure 1). Sample sizes and species names are detailed in Additional file 1. All specimens were identified as adults based on the ossification of the basisphenoid region. Mandible images were obtained with a digital camera Nikon Coolpix 8700. All specimens were photographed under the same plane in relation to the camera lens (reference points in the specimens were used to align the mandible with the focal plane). To capture as much shape variation as possible, 11 landmarks, and 25 semilandmarks were used (Figure 2). The reference points were digitalised in the TpsDig software [76]. The landmark configurations were superimposed according to a least squares criterion (Procrustes superimposition) [77-79], first calculating a mean for each species and then superimposing all configurations to calculate a grand average. The semilandmarks were slid to minimize the least squares criterion [80] in TPSRelw [81]. Even though slid semilandmarks do not hold biological correspondence (just like type II or III landmarks), there is a gain in the information on shape variation (as ascertained from a comparison of superimposed warped images in the program TpsSuper [82] - results not shown), and the correlation of Procrustes distance matrices obtained from data sets with and without semilandmarks was 0.956. After Procrustes superimposition, the aligned coordinates present more dimensions than the actual shape space (aside from the degrees of freedom lost in superimposition, a maximum of 48 dimensions is needed to describe 49 species means). To reduce dimensionality, the aligned coordinates were transformed to shape principal components [78]. Principal component analyses of aligned coordinates and the visualisation of shape changes associated with each principal component (shape PC) axis were performed in MorphoJ (importing the aligned landmarks and semilandmarks from TPSRelw) [35]. We have used Horn's parallel analysis [60,61] to choose the number of principal components to be retained. Parallel analysis is a method of consensus in the literature [61], and consists of generating a large number of random samples with the same number of variables (but uncorrelated) and specimens as the original data, and comparing the observed eigenvalues to the distribution of random eigenvalues. The observed PCs that presented eigenvalues larger than a percentile (we used 95%) of the random eigenvalue distribution are retained. We used the paran package [83] in the R environment [84] to perform the parallel analysis. Size was measured both as the centroid size (square root of summed squared distances from each landmark to the centroid of points) from mandible landmark configurations [77], and as Condylobasal Length (CL - from the anteriormost point on the premaxilla to the condyle). These two measures of size were highly correlated (*r *= 0.983), and all results were the same for both variables. Because cranial length is a more intuitive measure, and to facilitate further comparisons, we chose to report results using CL instead of mandible centroid size.

Dietary information was obtained from the literature (modified and updated from [5]). We organized the quantitative and qualitative information for the 49 bat species into five categories comprising the main food items identified in phyllostomid diets (insectivory [arthropods], carnivory [small vertebrates], frugivory, nectarivory [pollen and nectar], and sanguivory). With the exception of sanguivore bats, all phyllostomid species present mixed diets, with varying levels of specialization and predominance of items from a given category. To compare the relative importance of structurally different items, such as nectar and arthropods in the diet of a single species is a challenging task. We defined rank categories based on relative usage of food items (0 - absent, 1 - complementary, 2 - predominant, 3 - strict), and a rank was attributed to each dietary item for each species (see Additional file 1). The distribution of diet variables along the phylogenetic tree used [33] is depicted in Figure 1. The diet variables were used later as independent variables in a multivariate regression model. Preliminary analyses indicated strong multicollinearity among the diet variables, mostly caused by a positive correlation between insectivory and carnivory (sometimes lumped as animalivory), and a negative correlation between frugivory and animalivory. To avoid multicollinearity, we calculated the principal components (PCs) of a correlation matrix among the diet variables [85]. Details of this analysis have been published elsewhere [24]. The former approach allows for testing gradual shape changes in species groups with mixed diet. However, diet was also analysed as a discrete variable, using the predominant (or strict) diet item as category (see model-based analyses below).

### Phylogenetic comparative analysis of ecomorphological associations

Shape variation among species was summarised by principal components (the major axes of shape variation). The shape PCs were fitted to a phylogeny by estimating ancestral character states (nodes) for each PC and plotting the scores for OTUs (operational taxonomic units - the species at tree tips) and HTUs (hypothetical taxonomic units - the nodes) along with the branches linking the tree nodes. Each OTU and tree branch was plotted with a colour according to the predominant diet (estimated ancestral diet for the branches). A maximum likelihood algorithm [86] was used in the estimation of ancestral states, using the function ace in the APE package for the R environment for statistical computing [87,84]. The phylogeny of Baker *et al*. (2003; 2010) [3,33] (Figure 1), based on mtDNA and RAG2 sequences, was used as a framework for all the comparative analyses performed. The branch lengths used were the divergence time estimates obtained from Baker *et al*. (2010) [3]. This phylogeny has been independently corroborated in a recent paper [4], although the time estimates are slightly different between the publications.

Principal components of species average shapes depict the major axes of variation among species in shape space. These linear combinations of the original superimposed coordinates ordinate species means according to gradients formed by unknown latent factors. Associations of PC scores with measured factors can be performed *a posteriori *to aid in the interpretation of ordinations. The linear associations between mandible shape and diet principal components were tested by multivariate regression (where each data point is a species average), using a phylogenetic generalized linear model (PGLS - [40,88]). Generalized least squares (GLS) models allow for the phylogenetic non-independence to be incorporated into the error structure as an among species covariance matrix. Unlike spatially organised data (which share a similar problem of non-independence), where geographic distances among samples can be measured with small error, the phylogenetic covariances depend on the phylogenetic hypothesis they derive from and the evolutionary model assumed for character change. When phylogenetic covariances are based on the sum of branch lengths from the root of the tree to the most recent ancestral for each pair of species, a Brownian model of evolution is assumed, where the expected phenotypic differences among OTUs are directly related to their distance to the last common ancestor [88]. The Brownian model of evolution has been increasingly considered an unlikely assumption for comparative methods (particularly when dealing with adaptive radiations [28,38,39]). We used the estimate of covariance structure proposed by Martins and Hansen [40], that results from an evolutionary model that incorporates stabilising selection as a constraint. According to this model, covariances among OTUs are determined as *V_ij _*= *γ *exp(-*αt_ij_*), where *α *is a parameter determining the magnitude of a restraining force or pull towards an optimum phenotype, and *γ *is a parameter loosely interpreted as the interspecific variance in equilibrium (due to the balance between selective and neutral evolutionary forces). This model causes the influence of a common ancestor to decrease exponentially with the sum of branch lengths leading to that ancestor, as a consequence of stabilising selection "erasing" the evolutionary history. The value of *α *was interactively estimated via maximum likelihood during model fitting, providing flexibility of evolutionary models assumed. For the multivariate regression, the phylogenetic covariance matrix was estimated via the APE package [87] and the multivariate model fit with NTSYS-PC [89]. The shape PCs were included as dependent variables and the first three diet PCs plus average cranial size (Condylobasal Length - CL) were included as independent variables. The partial correlation coefficients from the multivariate regression were used to produce biplots showing the species ordination within the shape PC space and the associated directions of variation of diet PC vectors.

### Patterns of phenotypic and ecological disparity through time

Adaptive radiations commonly generate a distinctive pattern of temporal change in disparity, where morphological divergence increases quickly in the first phase, and slows down towards present time, as the niches are filled [22]. The change in disparity patterns through time (DTT) is usually studied by the comparison of fossil taxa [90]. However, Harmon and colleagues [91] have proposed and implemented (in the geiger package for the R environment [92]) a method to evaluate the changes in disparity patterns along a phylogenetic tree, using exclusively phenotypic measurements of terminal taxa. Using the full space of shape principal components (including all components with non zero eigenvalues) for the mandible shape, disparity was calculated from average pairwise Euclidean distances between species [91]. For this data set, this is equivalent to using average pairwise Procrustes distances (summed squared distances among corresponding landmarks after Procrustes superimposition) [79]. Using the full shape space is possible in this case because the data are reduced to pairwise distances regardless of dimensionality, and no parameters are estimated. However, the number of dimensions might have an effect in the simulations described below and the confidence intervals calculated, so it is more consistent to work with the full shape space. Size disparity was calculated using the same procedure, but using average Condylobasal Length as the single variable. Diet disparity was calculated as well, using the relative importance of dietary items (see Additional file 1) as data and average pairwise Manhattan distances as measure of disparity. Disparity through time (DTT) plots can be generated by first calculating the disparities for the entire tree and for each subclade defined by a node in the phylogeny. Relative disparities are obtained for each node by dividing its disparity by the tree disparity. Moving up from the root of the phylogeny, a mean disparity is calculated at each node (divergence event) as the average of the relative disparities of all lineages whose ancestral lineages were present at that point [91]. The DTT plots allow for insight on the relative timing of phenotypic divergence while avoiding the need of ancestral character estimation. The inference of disparity deviations from a neutral evolution pattern was performed by a comparison of observed patterns with the average of 1000 simulated DTT patterns after evolving the shape variables according to Brownian motion along the phylogenetic tree [91,93]. These simulations generate random phenotypes as phylogenetic independent contrasts along the tree (for each node) using a multivariate normal distribution with zero means and a covariance matrix based on the observed phylogenetic independent contrasts. A disparity distribution based on a neutral evolutionary process is then generated for each relative time (node) of the tree and the observed disparity can be compared to neutral confidence intervals.

A second approach to the investigation of evolutionary processes via a comparison of shape variation and relative time was based on Polly (2004) [34] simulation study. This author showed that different evolutionary models leave distinctive imprints on the distribution of shape distances versus time of divergence. We have compared the pairwise shape distances (Procrustes distances) to pairwise genetic distances (proportional to percent sequence divergence from Baker *et al*. (2003) [33]), and estimated time since divergence (from Baker *et al*. (2010) [3]) in a matrix scatterplot. This method allowed for a qualitative comparison of the observed pattern to the expected under different evolutionary modes by visual inspection [34].

### Modelling phenotypic evolution

The comparative methods based on GLS described above provide a direct test of linear relationships among major axes of diet and shape variation. Whereas this class of comparative methods might provide a statistically valid test of association, it provides no direct insight into the evolutionary processes responsible for the observed variation. A more recent approach, based on the simultaneous fit and comparison of evolutionary hypotheses combining evolutionary models, phenotypic and ecological information has been proposed by Butler and King (2004) [27] after Hansen's (1997) [94] method for the estimation of selective factors on adaptive optima (see also [28]). The model-based approach fits distributions predicted under a number of evolutionary hypothesis to observed data, allowing for simultaneous inference of model likelihood using information criteria.

The simplest model of phenotypic evolution is the neutral Brownian motion [25], where total evolutionary change in a continuous character is distributed normally, with a mean equal to the ancestral value for the entire tree and variance proportional to the amount of time elapsed from tree root to tips. An alternative evolutionary model (or class of models) for phenotypic change under stabilising selection that has gained popularity recently is based on the Ornstein-Uhlenbeck (O-U) process [27,28,40,94]. These models incorporate a constraint as a constant pulling force (measured by a parameter *α*) towards a central point (*θ*) which can mimic the action of stabilising selection on the phenotype. The central point can be interpreted as an adaptive optimum. In the approach proposed by Butler and King [27], the O-U models can have any number of adaptive optima (*θ_i_*), which are proposed according to hypotheses of ecological diversification. The models then incorporate a specific evolutionary process, a phylogeny with branch lengths (assumed to be at least proportional to time), and the selective regimes operative in each branch (derived from ancestral estimation of ecological changes).

All dietary ancestral states (branch colours in Figure 6) were estimated using the maximum likelihood algorithm implemented in the function ace in the APE package for the R environment [87]. The ancestral states and the terminal states form a design matrix that determines for each lineage leading to a particular terminal branch which optima where influential and the length of influence period. For example, in the lineage leading to *Glyphonycteris *in model OU.5 in Figure 6, the ancestors corresponding to the earlier branches were insectivores (for 8 My), then they are postulated to have switched to frugivory for around 5 My and switched back to insectivory for the remaining 17 My. The estimated phenotypic mean vector for *Glyphonycteris *would be a weighted sum of optima (*θ_i_*) leading to the terminal branch (see equation 5 in [27]). The weights determine that the most influential optima will be the ones closest to the terminal branch, and the influence reduces exponentially for optima that influenced a lineage near the root. In the case of *Glyphonycteris*, the most influential optimum would be insectivory (because of the terminal branch optimum, not the root), then frugivory. The optima for nectarivory, sanguivory and carnivory would have weight = 0 because there is no postulated *Glyphonycteris *ancestor with these optima. The models were fitted to the mandible shape data (the first three and five shape PCs) in a multivariate fashion and to cranial size (Condylobasal Length - CL). We used different numbers of shape PCs to assess the sensitivity of the models to the dimensionality of the subspace, changing both the number of parameters being estimated (see below) and the amount of variation being explained by the subspaces. Five PCs were indicated by parallel analysis to have larger eigenvalues than the random distribution of eigenvalues, whereas three PCs provide an optimal (maximum variance) visualisation of the shape subspace. In the Hansen model [27,94,95], the evolution of a multivariate phenotype with *p *variables assumes the form of the stochastic differential equation

where **A **is a *p *× *p *square symmetric matrix of *α *parameters that measure the strength of selection. the vector **q **is the optimum corresponding to the *θ_i _*for the particular branch, **S **is a *p *× *p *square symmetric matrix with sigma parameters that can be interpreted as the strength of random drift, and *B*(*t*) is a standard Wiener (Brownian motion) process [95]. The alpha and sigma matrices have each *p *× (*p *+ 1)/2 independent parameters (one for each variable and one for each pair of variables). These are estimated via an iterative optimisation procedure (we used identity matrices as starting forms) minimising the error of a multivariate GLS model that uses the alpha and sigma matrices to estimate the vector of optima **q **and the expected multivariate phenotype for each species (terminal branches) in the tree. Each model has to estimate 2 × *p *× (*p *+ 1)/2 + *p *× *n_θ _*parameters, where *n_θ _*is the number of optima in the model. Because matrices of alpha and sigma parameters are calculated, the models do not constrain selection strength and the random drift disturbances to be isotropic in shape space. On the other hand, a simplification is accomplished because the same alpha matrix is used for all postulated adaptive optima, requiring the selective constraints to be the same for all peaks. This is true also for univariate models because they use a single *α *parameter for all adaptive peaks [27].

The ecological (dietary) information available allowed for the elaboration of five alternative models of evolutionary change (BM, OU.2, OU.3, OU.4, OU.5). The first model only incorporates neutral evolution expectations along the phylogenetic tree according to a Brownian motion (BM) process. The other models use the O-U process with a number of different optima (Figure 6) according to postulated selective regimes. We start with a simple discrimination of two adaptive optima related to high and low mastication regimes (Model OU.2). Model OU.3 breaks the low mastication selective regimes into nectarivory and sanguivory, recognising the fundamental ecological and functional differences between them. Model OU.4 separates all dietary optima, but lumps insectivory and carnivory together into the animalivory category. A justification for this is the strong correlation between insectivory and carnivory observed in the diet PCA [24]. It has been suggested that carnivory is just an allometric extension of insectivory [36] and that these two categories share the same adaptive peak [24]. Model OU.5 discriminates adaptive optima for five selective regimes associated with each dietary category, representing the most complex hypothesis about the adaptive radiation of phyllostomid bats.

The models were compared by information loss criteria [59], such as the Akaike Information Criterion corrected for sample size (AICc) [29] and the more conservative Schwarz or Bayesian information criterion (SIC or BIC) [27]. The Information criteria were first transformed into differences from the minimum observed AICc value Δ*_i _*(AICc) = AICc_*i *_- min AICc. The differences are then transformed into AICc weights

for which ∑*w_i _*(AICc) = 1. The Akaike weight for a particular model *M_i _*can be interpreted as the probability of *M_i _*being the "best" model given the data and the set of candidate models [59]. The same procedure was applied for the SIC to calculate SIC weights. The Schwarz information criterion is more conservative than the AICc, because it imposes larger penalties for more complex models. The SIC also assumes that the true model is within the candidate set of models, and is low dimensional, whereas the AICc assumes that the true model is not within the candidate set and is infinitely dimensional [59]. Confidence limits (ellipsoids) for adaptive optima were estimated by a parametric bootstrap (resampled 5000 times). All model fitting procedures were performed using package OUCH (version 2.7-1) [95] for the R environment.

## Authors' contributions

Both authors contributed to the design of the study. MRN collected the shape data (travelled to Museums, photographed specimens and digitized landmarks) and assembled the diet data from the literature. Both authors performed the analyses. LRM wrote a first draft with contributions from MRN. Both authors read and approved the final manuscript.

## Supplementary Material

Additional file 1 **Table of sample sizes, diet and shape variables per species**. File in pdf format with sample sizes, dietary preferences, cranial size and shape principal component scores for each species. Click here for file

Additional file 2 **Animation showing the principal component ordination of shape**. File in gif format (animated gif) showing a rotating shape principal component ordination with species names. Click here for file
