# Supplementary material for: Evolutionary patterns and processes in the radiation of phyllostomid bats
Source: BMC Evol Biol. 2011 May 23;11:137. doi: 10.1186/1471-2148-11-137 (PMC3130678; doi:10.1186/1471-2148-11-137)
Supplement: Additional file 1 — Table of sample sizes, diet and shape variables per species. File in pdf format with sample sizes, dietary preferences, cranial size and shape principal component scores for each species. [file 1471-2148-11-137-S1.PDF]

List of species studied, with sample sizes (N) and rank dietary variables (modified from Wetterer et al. 2000. *B. Am. Mus. Nat. Hist.* 248:1-200; and Monteiro and Nogueira 2010. *Evolution* 64:724-744). Rank categories are based on relative usage of food items (0 - absent, 1 - complementary, 2 - predominant, 3 - strict)

Species	N	Insectivory	Carnivory	Sanguivory	Frugivory	Nectarivory
Ametrida centurio	2	0	0	0	3	0
Sphaeronycteris toxophyllum	2	0	0	0	3	0
Pygoderma bilabiatum	13	0	0	0	3	0
Centurio senex	5	0	0	0	3	0
Stenoderma rufum	1	0	0	0	3	0
Ariteus flavescens	1	0	0	0	3	0
Artibeus obscurus	20	1	0	0	2	1
Dermanura cinerea	1	0	0	0	3	0
Enchisthenes hartii	5	0	0	0	3	0
Platyrrhinus lineatus	20	1	0	0	2	1
Vampyrodes caraccioli	29	1	0	0	2	1
Mesophylla macconnelli	11	1	0	0	2	1
Vampyressa pusilla	12	1	0	0	2	1
Uroderma magnirostrum	13	1	0	0	2	1
Vampyriscus bidens	6	1	0	0	2	1
Chiroderma doriae	13	1	0	0	2	1
Sturnira lilium	18	1	0	0	2	1
Rhynophylla pumilio	12	1	0	0	2	1
Glyphonycteris sylvestris	3	2	0	0	1	0
Trinycteris nicefori	9	2	0	0	1	0
Carollia perspicillata	14	1	0	0	2	1
Lonchophylla thomasi	6	1	0	0	1	2
Lionycteris spurrelli	10	1	0	0	1	2
Glossophaga soricina	10	1	0	0	1	2
Leptonycteris nivalis	10	1	0	0	1	2
Monophyllus redmani	10	1	0	0	1	2
Brachyphylla cavernarum	11	1	0	0	1	2
Erophylla sezekorni	10	1	0	0	1	2
Musonycteris harrisoni	1	1	0	0	1	2
Choeronycteris mexicana	10	1	0	0	1	2
Choeroniscus minor	9	1	0	0	1	2
Hylonycteris underwoodi	4	1	0	0	1	2
Anoura caudifer	11	1	0	0	1	2
Mimon crenulatum	11	2	1	0	1	0
Phyllostomus hastatus	3	2	1	0	1	1
Phylloderma stenops	2	2	0	0	1	1
Tonatia saurophila	7	2	1	0	1	0
Lophostoma brasiliense	9	2	0	0	1	0
Chrotopterus auritus	11	1	2	0	1	0
Vampyrum spectrum	7	1	2	0	1	0
Macrophyllum macrophyllum	6	3	0	0	0	0
Trachops cirrhosus	13	2	2	0	1	0
Lonchorhina aurita	14	3	0	0	1	0
Desmodus rotundus	10	0	0	3	0	0
Diaemus youngi	2	0	0	3	0	0
Diphylla ecaudata	12	0	0	3	0	0
Micronycteris minuta	12	2	0	0	1	0
Lampronycteris brachyotis	2	2	0	0	1	0
Macrotus waterhousii	10	2	0	0	1	0

Average Basicranial Skull Length (BSL) and Diet PC scores . DietPC1 is positively correlated with Insectivory (0.811) and Carnivory (0.533), and negatively correlated with Frugivory (-0.834). Diet PC2 is positively correlated with Sanguivory (0.982), and negatively correlated with Insectivory (0.428), Frugivory (0.340) and Nectarivory (0.364). Diet PC3 is positively correlated with Nectarivory (0.826) and negatively correlated with Carnivory (-0.614) and Frugivory (-0.418).

Species	BSL (mm)	DietPC1	DietPC2	DietPC3
Ametrida centurio	10.78	-0.873	-0.386	-0.500
Sphaeronycteris toxophyllum	11.93	-0.873	-0.386	-0.500
Pygoderma bilabiatum	15.86	-0.873	-0.386	-0.500
Centurio senex	11.87	-0.873	-0.386	-0.500
Stenoderma rufum	16.58	-0.873	-0.386	-0.500
Ariteus flavescens	13.51	-0.873	-0.386	-0.500
Artibeus obscurus	23.65	-0.318	-0.557	0.003
Dermanura cinerea	15.27	-0.873	-0.386	-0.500
Enchisthenes hartii	17.07	-0.873	-0.386	-0.500
Platyrrhinus lineatus	19.91	-0.318	-0.557	0.003
Vampyrodes caraccioli	19.26	-0.318	-0.557	0.003
Mesophylla macconnelli	13.68	-0.318	-0.557	0.003
Vampyressa pusilla	16.64	-0.318	-0.557	0.003
Uroderma magnirostrum	20.49	-0.318	-0.557	0.003
Vampyriscus bidens	16.24	-0.318	-0.557	0.003
Chiroderma doriae	24.51	-0.318	-0.557	0.003
Sturnira lilium	18.75	-0.318	-0.557	0.003
Rhynophylla pumilio	15.14	-0.318	-0.557	0.003
Glyphonycteris sylvestris	16.99	0.275	-0.452	-0.153
Trinycteris nicefori	17.54	0.275	-0.452	-0.153
Carollia perspicillata	18.28	-0.318	-0.557	0.003
Lonchophylla thomasi	18.54	-0.046	-0.566	0.499
Lionycteris spurrelli	17.07	-0.046	-0.566	0.499
Glossophaga soricina	18.2	-0.046	-0.566	0.499
Leptonycteris nivalis	23.6	-0.046	-0.566	0.499
Monophyllus redmani	19.69	-0.046	-0.566	0.499
Brachyphylla cavernarum	26.06	-0.046	-0.566	0.499
Erophylla sezekorni	20.4	-0.046	-0.566	0.499
Musonycteris harrisoni	31.08	-0.046	-0.566	0.499
Choeronycteris mexicana	26.78	-0.046	-0.566	0.499
Choeroniscus minor	20.97	-0.046	-0.566	0.499
Hylonycteris underwoodi	18.93	-0.046	-0.566	0.499
Anoura caudifer	19.98	-0.046	-0.566	0.499
Mimon crenulatum	17.03	0.460	-0.466	-0.398
Phyllostomus hastatus	33.38	0.442	-0.604	-0.069
Phylloderma stenops	27.96	0.256	-0.590	0.177
Tonatia saurophila	22.19	0.460	-0.466	-0.398
Lophostoma brasiliense	15.74	0.275	-0.452	-0.153
Chrotopterus auritus	31.24	0.363	-0.317	-0.650
Vampyrum spectrum	41.67	0.363	-0.317	-0.650
Macrophyllum macrophyllum	13.09	0.849	-0.486	0.020
Trachops cirrhosus	25.04	0.646	-0.479	-0.643
Lonchorhina aurita	15.99	0.558	-0.614	-0.146
Desmodus rotundus	20.92	0.022	0.372	0.059
Diaemus youngi	18.54	0.022	0.372	0.059
Diphylla ecaudata	18.88	0.022	0.372	0.059
Micronycteris minuta	15.07	0.275	-0.452	-0.153
Lampronycteris brachyotis	17.75	0.275	-0.452	-0.153
Macrotus waterhousii	19.62	0.275	-0.452	-0.153

Shape PC scores used in the analyses.

Species	ShPC1	ShPC2	ShPC3	ShPC4	ShPC5
Ametrida centurio	-0.060	0.054	-0.044	0.042	-0.008
Sphaeronycteris toxophyllum	-0.082	0.025	-0.035	0.042	-0.017
Pygoderma bilabiatum	-0.071	0.004	-0.042	0.064	-0.032
Centurio senex	-0.018	0.001	0.019	0.085	-0.038
Stenoderma rufum	-0.126	0.030	-0.007	0.055	0.028
Ariteus flavescens	-0.103	0.026	-0.018	0.052	0.024
Artibeus obscurus	-0.061	0.028	-0.002	0.002	0.001
Dermanura cinerea	-0.041	0.030	-0.028	0.008	-0.001
Enchisthenes hartii	-0.023	0.015	-0.036	-0.004	-0.002
Platyrrhinus lineatus	-0.021	0.034	-0.033	-0.024	-0.017
Vampyrodes caraccioli	-0.084	0.049	-0.007	0.001	0.021
Mesophylla macconnelli	0.028	0.050	0.007	-0.003	-0.030
Vampyressa pusilla	-0.011	-0.003	-0.043	-0.003	-0.032
Uroderma magnirostrum	0.004	0.020	-0.031	-0.015	-0.029
Vampyriscus bidens	-0.029	0.006	-0.031	-0.023	-0.031
Chiroderma doriae	-0.049	0.028	-0.011	-0.015	0.004
Sturnira lilium	-0.051	-0.013	-0.017	-0.011	-0.012
Rhynophylla pumilio	-0.006	-0.044	-0.044	-0.033	-0.017
Glyphonycteris sylvestris	0.049	0.055	0.053	0.002	0.017
Trinycteris nicefori	0.014	0.029	0.004	-0.016	0.010
Carollia perspicillata	-0.009	0.018	-0.038	-0.012	0.002
Lonchophylla thomasi	0.088	-0.041	0.009	0.007	-0.017
Lionycteris spurrelli	0.094	-0.040	-0.014	-0.001	0.018
Glossophaga soricina	0.096	-0.019	0.026	0.020	0.008
Leptonycteris nivalis	0.118	-0.065	0.014	0.010	-0.007
Monophyllus redmani	0.116	-0.018	0.040	0.018	-0.019
Brachyphylla cavernarum	-0.056	-0.018	-0.003	-0.023	0.051
Erophylla sezekorni	0.049	-0.001	-0.027	-0.036	-0.007
Musonycteris harrisoni	0.200	-0.022	-0.080	-0.010	0.015
Choeronycteris mexicana	0.168	-0.014	-0.037	0.000	0.004
Choeroniscus minor	0.179	-0.058	-0.019	0.018	0.022
Hylonycteris underwoodi	0.114	-0.033	-0.005	0.009	0.018
Anoura caudifer	0.107	-0.029	0.034	0.025	-0.010
Mimon crenulatum	0.011	0.056	0.015	-0.008	0.011
Phyllostomus hastatus	-0.034	0.067	0.011	-0.019	0.021
Phylloderma stenops	-0.063	-0.028	-0.006	-0.034	0.025
Tonatia saurophila	-0.069	0.027	0.012	-0.025	0.012
Lophostoma brasiliense	-0.059	0.042	0.020	-0.033	0.009
Chrotopterus auritus	-0.042	0.018	0.027	-0.050	-0.017
Vampyrum spectrum	-0.023	0.030	0.035	-0.065	-0.059
Macrophyllum macrophyllum	0.004	0.053	0.028	0.007	0.037
Trachops cirrhosus	0.027	-0.002	0.098	0.010	-0.021
Lonchorhina aurita	0.044	0.028	0.026	0.004	0.019
Desmodus rotundus	-0.134	-0.198	0.076	0.007	-0.018
Diaemus youngi	-0.135	-0.156	-0.019	-0.014	0.021
Diphylla ecaudata	-0.084	-0.159	-0.013	-0.010	0.016
Micronycteris minuta	0.031	0.044	0.039	-0.006	-0.001
Lampronycteris brachyotis	0.001	0.032	0.056	0.013	0.017
Macrotus waterhousii	0.003	0.060	0.042	-0.007	0.010
