# Supplementary figures and images for: Evolutionary patterns and processes in the radiation of phyllostomid bats
Source: BMC Evol Biol. 2011 May 23;11:137. doi: 10.1186/1471-2148-11-137 (PMC3130678; doi:10.1186/1471-2148-11-137)

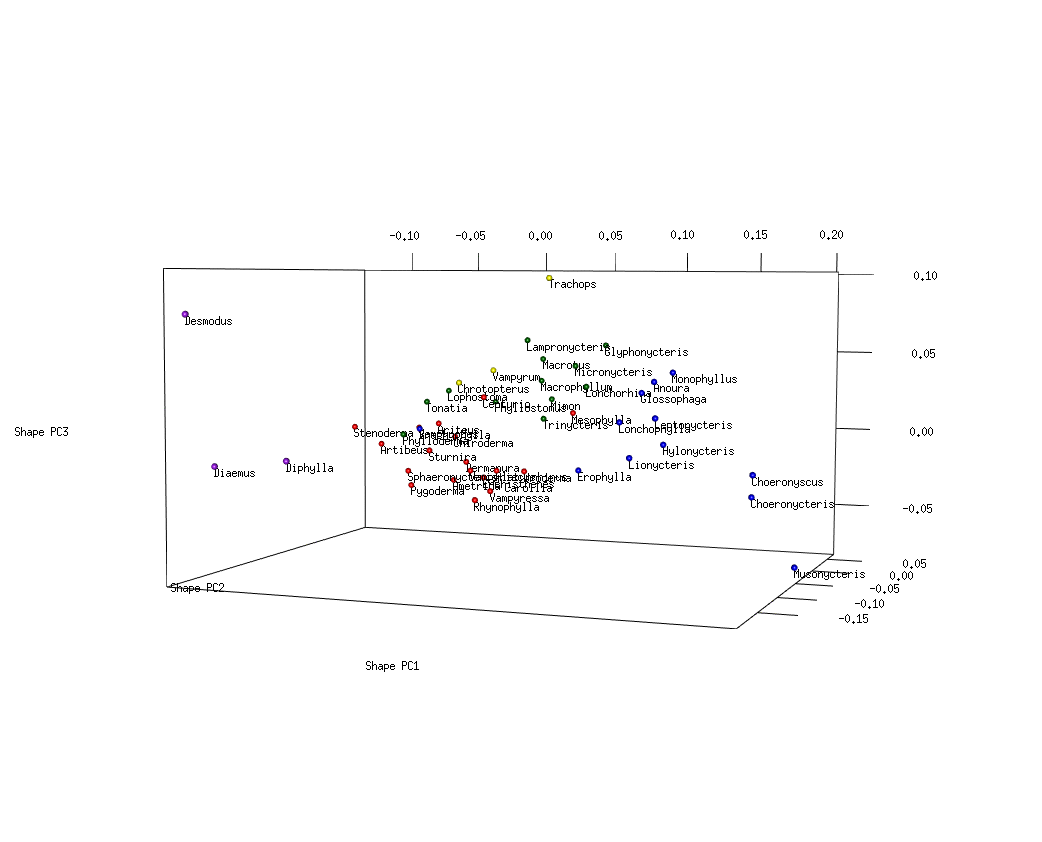

Supplement: Additional file 2 — Animation showing the principal component ordination of shape. File in gif format (animated gif) showing a rotating shape principal component ordination with species names. [file 1471-2148-11-137-S2.GIF]
